# The theory of island biogeography and the stability of oceanic reef fish communities

**DOI:** 10.1111/jfb.70367

**Published:** 2026-02-22

**Authors:** Esteban Jorcin Nogueira, Camille Mellin, Carlos E. L. Ferreira, Hudson Tercio Pinheiro

**Affiliations:** ^1^ Department of Zoology, Institute of Biological Sciences University of São Paulo São Sebastião Brazil; ^2^ Post‐graduate Program in Zoology, Institute of Biosciences University of São Paulo São Paulo Brazil; ^3^ The Environment Institute and School of Biological Sciences University of Adelaide Adelaide South Australia Australia; ^4^ Fluminense Federal University Niteroi Brazil

**Keywords:** ecological monitoring, geographic isolation, oceanic islands, population dynamics, reef fish, temporal analyses

## Abstract

Reef fish assemblages on oceanic islands offer valuable insights into ecological and evolutionary processes, yet their temporal dynamics remain poorly understood. This study evaluates the theory of island biogeography (TIB) with long‐term ecological monitoring to assess how island area, isolation from nearest reef and distance from mainland shape both species richness and stability across four southwestern Atlantic islands. Data were collected via underwater visual censuses from 2006 to 2011 and annually thereafter. A total of 3634 transects were conducted, recording over 150 fish species. Spatial β diversity was quantified using Sørensen and Bray–Curtis dissimilarity indices. Results indicate that larger islands, with greater habitat heterogeneity, support higher species richness and exhibit increased assemblage dissimilarity but reduced variability, suggesting a buffering effect against compositional shifts. Temporal analyses revealed that species turnover is the primary driver of annual changes in assemblage composition, with distinct patterns emerging according to island size. Isolation is significantly associated with lower temporal β diversity, whereas larger reef areas promote more dynamic shifts in assemblage structure. These findings provide empirical support for TIB, emphasizing that both island area and isolation are key factors influencing the richness and stability of reef fish assemblages. Long‐term monitoring, although scarce in the marine realm, is essential for capturing these complex dynamics and informing conservation strategies in the face of increasing anthropogenic pressures on unique insular marine ecosystems.

## 1 INTRODUCTION

Reef ecosystems are among the most diverse environments globally, harbouring the greatest numbers of species in the marine realm (Bellwood & Hughes, 2001; Spalding et al., 2001). In addition to the complexity of coastal reefs, a diversity of reef environments occurs around oceanic islands, which serve as natural laboratories for understanding ecological and evolutionary processes. Oceanic islands, formed by volcanic or tectonic processes, have never been connected to the continents (Dawson, 2015). Their geographic isolation, unique geological histories and sensitivity to sea‐level fluctuations have resulted in distinct biogeographical patterns that influence assemblage structure and temporal stability in ways that differ from those observed in continental and coastal environments (Fernández‐Palacios, 2016; Pinheiro et al., 2017).

A fundamental aspect of oceanic islands is their patterns of species richness and endemism, which are largely driven by isolation and limited connectivity with larger biogeographic regions. These factors typically result in lower overall species richness but can promote high rates of speciation and favour greater proportions of endemic species (Losos & Ricklefs, 2009; Serafini et al., 2010). The classic *Theory of Island Biogeography* (MacArthur & Wilson, 1967) provides a foundation for understanding these patterns, demonstrating that the balance between colonization and extinction processes, shaped by both species traits and island geography, determines the insular biodiversity through a dynamic equilibrium (Hachich et al., 2020; MacArthur & Wilson, 1963, 1967; Paulay, 1994). Some species exhibit high colonization rates, arriving early and frequently, whereas others, due to lower dispersal capabilities, reach the islands later and face greater risks of extinction (MacArthur & Wilson, 1963, 1967), a pattern that also fosters speciation and high endemism in oceanic islands (Pinheiro et al., 2017). Consequently, over time, smaller and more isolated islands tend to harbour fewer species, whereas larger and more connected islands support richer communities, creating a dynamic equilibrium that might govern the stability of insular assemblages. However, how this dynamic equilibrium works through ecological time scales is still understudied. It is known that smaller and isolated coral reefs are more susceptible to fluctuations in composition, and thus, community stability is strongly correlated with reef size and isolation (McCann, 2000; Mellin et al., 2010).

Despite significant advances in island biogeography, the temporal dynamics of reef fish communities in oceanic islands remain poorly understood (Dawson, 2015; Magurran et al., 2019; Quimbayo et al., 2019). Long‐term studies are critical for discerning trends in community stability and for elucidating how geographic factors influence the natural variation in insular biodiversity. Understanding these dynamics is essential not only for advancing ecological theory but also for enhancing conservation strategies in the face of escalating anthropogenic pressures. Oceanic islands are among the last marine refuges, but even these remote and unpopulated islands are not immune to human impacts (Hughes et al., 2017). Many are increasingly being affected by human‐induced disturbances such as overfishing, habitat degradation and climate change, which can amplify species invasions and extinction rates (Cinner et al., 2016; Mellin et al., 2016; Sala et al., 2021; Sandin et al., 2008). Those already subject to natural fluctuations and species turnover may be particularly vulnerable to additional stressors.

In the southwestern Atlantic, four major oceanic islands provide an exceptional setting for testing biogeographical hypotheses: Trindade Island, Rocas Atoll, St. Peter and St. Paul (SPSP) Archipelago and Fernando de Noronha Archipelago (Floeter et al., 2007). These insular environments differ significantly in island size, distance from the mainland and isolation from the nearest reef, offering a robust comparative framework to evaluate how geographic factors and the theory of island biogeography (TIB) shape both richness and stability of reef fish assemblages. Larger islands, such as Fernando de Noronha (located closer to the coast) and Trindade (situated farther), host higher local species richness, whereas smaller and more isolated islands, such as SPSP, exhibit higher levels of endemism and yield more restricted species pools (Pinheiro et al., 2018; Quimbayo et al., 2019).

This study aims to investigate the relationship between island biogeography theory and assemblage stability, by exploring the long‐term temporal and spatial dynamics of reef fish assemblages across the southwestern Atlantic islands. We assessed species composition, richness and abundance variation using β diversity and Bray–Curtis metrics to evaluate spatial and temporal assemblage dissimilarities (Baselga, 2009; Dornelas et al., 2014). By incorporating island area, isolation and distance from the mainland into our analysis, we tested whether assemblage stability conforms to the predictions of the TIB (MacArthur & Wilson, 1963, 1967) and aligns with established patterns in reef ecosystem stability (Mellin et al., 2010). Thus, we hypothesize that reef fish assemblages on smaller and more isolated islands, such as SPSP, will exhibit greater temporal variation, whereas those on larger islands will demonstrate enhanced stability over time. Our findings provide new insights into the resilience of reef fish assemblages in oceanic islands, underscoring the role of biogeographical processes in shaping composition and stability across both spatial and temporal scales.

## 2 MATERIALS AND METHODS

### 2.1 Study area and data collection

**FIGURE 1:**
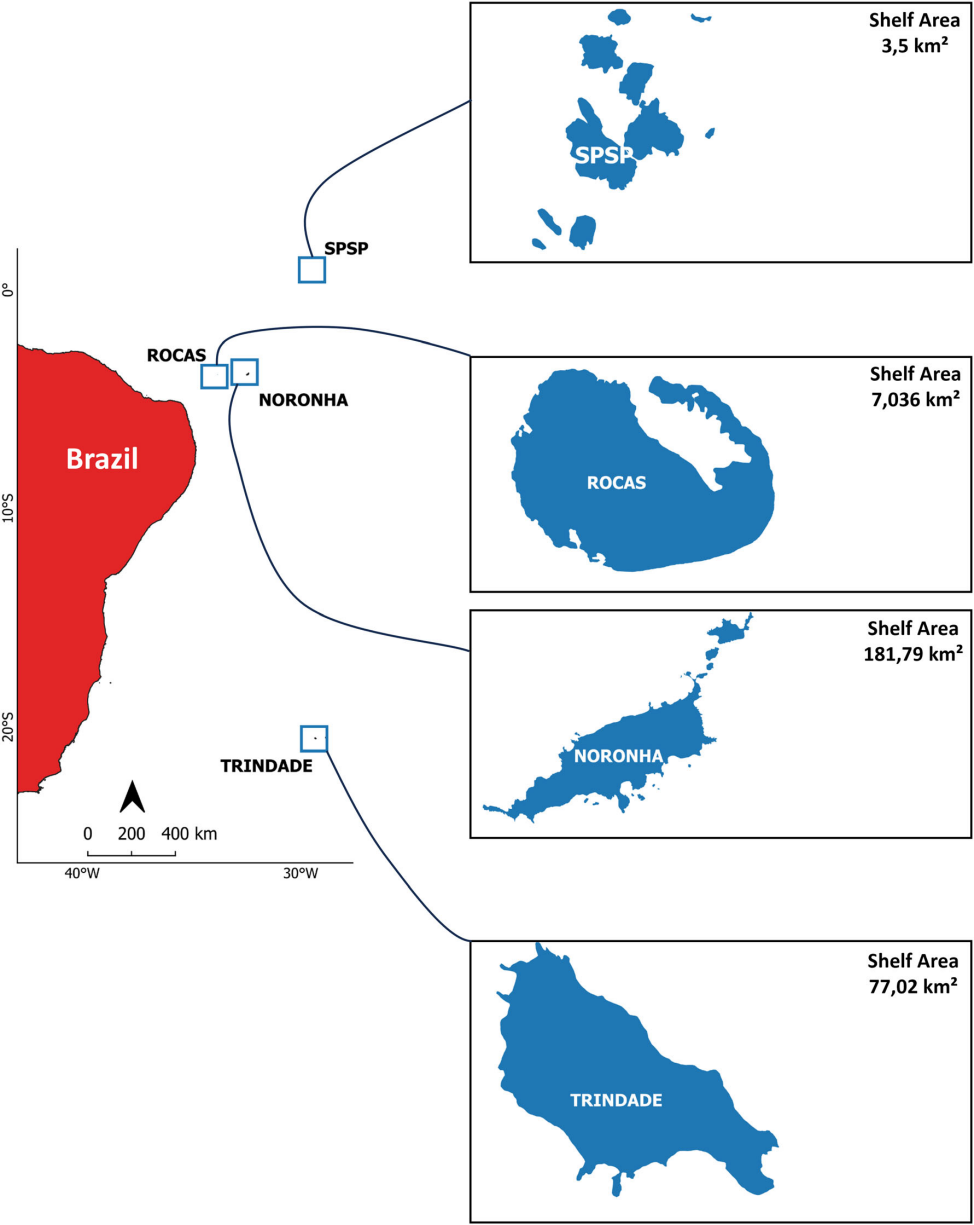
Map of Brazil with the respective oceanic islands studied: red, the continent; blue, the analysed islands: SPSP (St. Peter and St. Paul Archipelago), Rocas (Rocas Atoll), Noronha (Fernando de Noronha) and Trindade Island.

Data were collected from the four oceanic islands of the southwestern Atlantic: Rocas Atoll (Rocas), Fernando de Noronha Archipelago (Noronha), SPSP Archipelago and Trindade Island (Trindade) (see Table 1 and Figure 1 for more details).

**TABLE 1:** Distance from the coast, isolation from the nearest reef, shelf area, average reef fish richness per transect, reef fish richness of the insular species pool (Quimbayo et al., 2019; Ferrari et al., 2023 adapted).

Island	Isolation to the nearest shallow reef (km)	Distance to mainland (km)	Shelf area (km^2^)	Richness per transect (Species per 40 m^2^)	Total richness
Rocas	145	288	7.04	10.74	123
Fernando de Noronha	145	345	181.80	11.09	151
Trindade	50	1150	77.02	11.02	154
SPSP	630	1010	3.50	9.92	59

Abbreviation: SPSP, St. Peter and St. Paul.

Data collection occurred in a discontinued manner between 2006 and 2011 (Rocas: 2006; Fernando de Noronha: 2007; SPSP: 2009 and 2011; Trindade: 2007, 2009 and 2011) and then annually at all islands since 2012, as part of the Programa Ecológico de Longa Duração nas Ilhas Oceânicas (PELD ILOC) programme (CNPq 441241/2016‐6, CELF‐PI) (https://peldiloc.sites.ufsc.br/). Although pre‐2012 data were sampled opportunistically, they provide important insights into community variation over the years.

Underwater visual censuses were conducted in reef environments at depths between 3 and 15 m, with strip transects made while free or scuba diving. In each census, observations were carried out along a 20‐m‐long transect (2 m wide), which resulted in a total sampled area of 40 m^2^. During these transects, the species were identified, the total number of individuals was recorded and their sizes were estimated to the nearest centimetre. Non‐cryptic fishes (>10 cm) were counted first, when divers unwounded the tape, whereas benthic‐associated non‐cryptic fishes and cryptic species were counted later, when divers were retracting the tape. For large schools, the number of individuals in a quarter of the school was estimated and multiplied by four. Because visibility was usually lower than the transect length, counts of species were not instantaneous but gradual at 3–6 m in front of the diver (depending on the visibility) (Morais et al., 2017). Data for environmental characterization were not acquired.

The study sites within each island were predefined and standardized at the beginning of the research programme. Monitoring yielded a record of 150 species (Table S1) in 3634 transects: Rocas had 75 species and 738 transects, Fernando de Noronha had 85 species and 889 transects, Trindade recorded 94 species and 1501 transects and SPSP had 47 species and 506 transects (Table 1).

### 2.2 Data analysis

To conduct a standardized spatial and temporal analysis of reef fish assemblages, we established the minimum sampling effort (consistent with coverage‐based standardization) required to determine fish composition for each island based on rarefaction curves (Chao & Jost, 2012; Gotelli & Colwell, 2011). This minimum effort was dictated by the islands with the lowest sampling intensity (23 transects totalling 920 m^2^ in SPSP), and subsequently, all islands were standardized to this minimum, thus excluding the sampling size effect (see Figure S1). Years that did not meet the sampling effort in the rarefaction curves for each individual island were excluded from the analysis (2011 and 2018, for Fernando de Noronha Archipelago, and 2016 for Trindade Island). After this standardization process, the dataset consisted of fish abundance matrices spanning 9 years for Fernando de Noronha Archipelago, 10 years for Rocas Atoll, 10 years for SPSP and 11 years for Trindade Island.

For spatial variation assessment, fish community composition at transect level was analysed for each island, whereas for temporal variation, we examined the mean species richness per year (using the minimum sampling effort, equivalent to an area of 920 m^2^). Changes in species composition and abundance among transects (spatial variation) and across years (temporal variation) were evaluated following the methods of Baselga (2009).

β Diversity, as measured using the Sørensen dissimilarity index, was decomposed into its two main components: turnover (using the Simpson index, representing species substitution) and nestedness (indicating when a less diverse community is a subset of a richer one). Similarly, the Bray–Curtis dissimilarity (which accounts for species abundance) was decomposed into balance (representing species abundance shifts in opposite directions by equal magnitude) and abundance gradient (reflecting independent changes in species abundances) (Baselga, 2013). To homogenize variance among samples, the species abundance data were log transformed. β Diversity values ranged from 0 (identical) to 1 (completely dissimilar). Both Sørensen and Bray–Curtis dissimilarity analyses were executed following Baselga and Orme (2012), package *betapart*.

Spatial analyses were based on the richness, and fish assemblage corresponded to the minimum sampling effort (23 transects, 920 m^2^). Because the number of transects available for each island differed in each year, we randomly resampled a fixed number of these transects (23 transects) to generate average dissimilarity values for each location/year based on 1000 permutations. This approach allowed the use of all transects sampled in locations (mainly larger islands) or years that presented more transects than the minimal sampling effort of SPSP comparing the observed communities in a standardized manner. Spatial analyses employed large numbers of dissimilarity values (253 transect‐level combinations per year per island), whereas temporal analysis yielded 36 comparisons for Fernando de Noronha, 45 for Rocas Atoll, 45 for SPSP and 55 for Trindade Island.

Six Kruskal–Wallis tests, followed by Dunn–Bonferroni post hoc comparisons (Dunn, 1961; Kruskal & Wallis, 1952), were used to assess significant differences in variance of spatial and temporal Sørensen and Bray–Curtis dissimilarities (and their components) as well as species richness across islands (see Table S2); additional Hellinger transformed data were used to compare the robustness of the original (see Table S8).

To further assess the influence of the TIB on these insular assemblages, we applied the generalized linear mixed effect models (GLMM), using logit‐transformed links and β distribution, to analyse patterns in spatial and temporal β diversity (using both Sørensen and Bray–Curtis indices as response variables). Predictor variables included distance to continent, isolation from the nearest reef and reef area, with island treated as a random factor. Alternatives GLMMs, with ‘island’ as a fixed term, using the logit‐transform and fitted Gaussian models were compared to the original models for robustness (Table S7). All predictors were standardized to a mean of 0 and a standard deviation of 1. Residuals were examined to ensure normality, and variance‐inflation factors (VIF) between variables were calculated (see Figure S3; Table S6). To account for temporal autocorrelation, we applied a block bootstrap approach (1000 resamples) stratified by year.

Non‐metric multidimensional scaling was performed using the Jaccard index applied to a presence–absence matrix to explore temporal variation in reef fish assemblages for each island, as this is a robust ordination method for community ecology (Minchin, 1987). A permutational multivariate analysis of variance (PERMANOVA) on distance matrices (adonis) and a test for homogeneity of multivariate dispersions (betadisper) (Table S4) were used to assess temporal differences in community structure within each island. Both tests were paired in the interpretation: PERMANOVA evaluated the differences in multivariate centroids among years, whereas the test for homogeneity assessed whether group dispersions differed significantly. Presence and absence maps, as well as abundance heat maps, were prepared for a better visualization of how the assemblages have been varying over the years in each island (Figure S2). All analyses were performed in R, version 4.4.1 (R Development Core Team, 2025). Further details on packages and scripts used can be found in the supplementary files.

### 2.3 Ethics statement

This research was authorized by ICMBio (SISBIO 41327‐45) and did not use any live animals in experiments.

## 3 RESULTS

**FIGURE 2:**
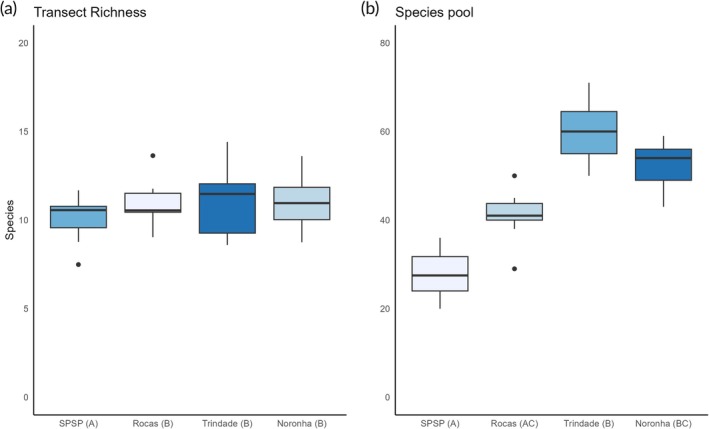
Box plot representing the small‐scale richness (transects) and its spatial variation, and the species pool determined by the minimal sampling effort and its temporal variation. Different letters indicate significant differences among islands (from the smallest to the biggest island), whereas the same letters indicate no difference. Panel “a” represents the Transect Richness, panel “b” represents the Species pool.

**FIGURE 3:**
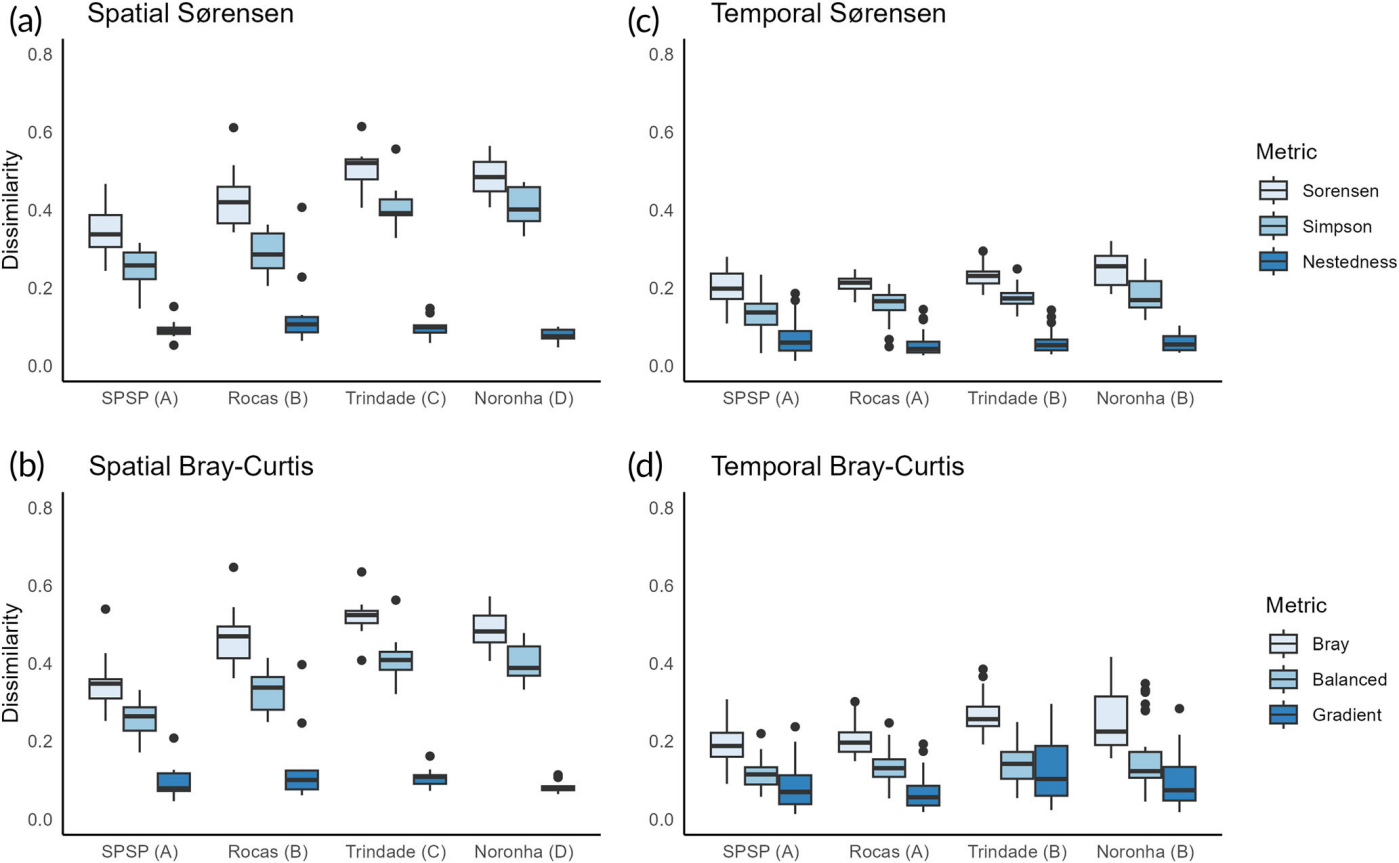
Box plot representing average of temporal and spatial Sørensen dissimilarity and its components (Simpson and nestedness), as well as Bray–Curtis and its components (balanced and gradient), for each island. Different letters indicate significant differences among islands, whereas the same letters indicate no difference. Panels “a and b” represents the Spatial Sørensen and Bray‐Curtis dissimilarity, while panels “c and d” represents the Temporal Sørensen and Bray‐Curtis dissimilarity.

Species richness per transect (small scale) was relatively similar among the islands, with exception of the smallest and most isolated island, which presented lowest values (Kruskal–Wallis with Dunn–Bonferroni, *p* < 0.05; Figure 2a). The richness of the species pool exhibited a similar trend, yet with greater variation among islands (Figure 2b). The highest values were found in the larger islands (e.g. Trindade ~60 species), whereas the lowest values occurred in the smallest and most isolated one (SPSP ~27.7 species), with Fernando de Noronha and Rocas having intermediate richness values (~52.5 and 41.1 species, respectively) (Figure 2b). The spatial variation in richness, differently, changed with the scale, where the highest coefficient of variation (CV) for transect richness was found in large islands, whereas the highest for the species pool was found in the smallest island (18% and 19%, respectively). The spatial dissimilarity of fish composition (Sørensen dissimilarity) and abundance (Bray–Curtis dissimilarity) had the highest values in the largest islands (Figure 3a,b; Table S2). Although presenting lower spatial dissimilarity in both indices, smaller islands exhibited the highest CVs (Table 2). The Sørensen spatial dissimilarity was primarily driven by the Simpson turnover rather than nestedness (representing between 68% and 84% of the total dissimilarity depending on the island), whereas the Bray–Curtis index, similarly, was mainly driven by the abundance balance instead of abundance gradients (representing between 70% and 83% of the total dissimilarity depending on the island) (Figure 3a,b; Table S5).

**TABLE 2:** Coefficient of variance for each island.

Island	Transect richness (spatial analysis) (%)	Species pool richness (temporal analysis) (%)	Spatial Sørensen (%)	Spatial Bray–Curtis (%)	Temporal Sørensen (%)	Temporal Bray–Curtis (%)
Rocas	10.9	13.2	19.2	18.2	9.2	18.3
Fernando de Noronha	11.8	9.5	10.2	12.2	17.0	29.8
SPSP	12.7	19.0	19.8	23.1	21.1	25.1
Trindade	18.0	10.9	10.2	10.4	12.8	16.5

Abbreviation: SPSP, St. Peter and St. Paul.

We found similar patterns in terms of temporal dissimilarity, although lower values were observed compared to the spatial patterns. The largest islands maintained the higher values in both Sørensen and Bray–Curtis dissimilarities compared to the smallest island (Figure 3c,d; Table S2).

**FIGURE 4:**
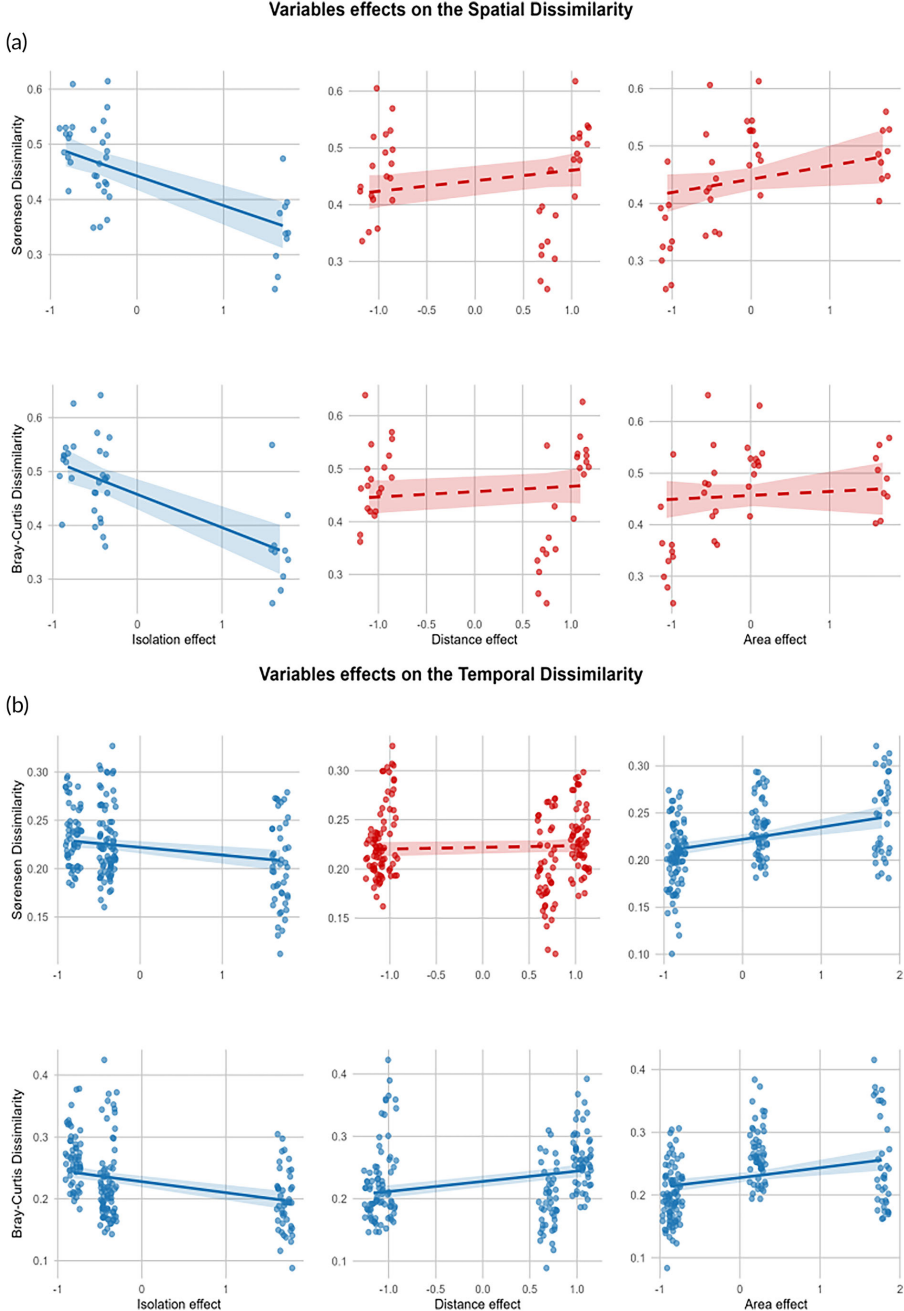
GLMM (generalized linear mixed effect model) of spatial and temporal dissimilarity; blue colour and solid line represent significant correlation; red colour and dashed line represent no significant correlation. Panel “a” relates to the Spatial Dissimilarity, panel “b” relates to the Temporal Dissimilarity.

The linear models (GLMM) indicated that isolation was the main driver of both spatial Sørensen (*Z*‐value: −4.435, *p* < 0.05) and Bray–Curtis dissimilarities (*Z*‐value: −4.664, *p* < 0.05), showing a negative relationship (Figure 4a). Therefore, more isolated islands were more compositionally homogeneous (Table S3).

The models assessing temporal dissimilarity exhibited similar patterns for both indices, with isolation being significantly and negatively correlated with the dissimilarity (Sørensen *Z*‐value: −2.7, *p* < 0.05, and Bray–Curtis *Z*‐value: −4.32, *p* < 0.05) (Figure 4b). However, area was also important, positively correlated with dissimilarity for both indices (Sørensen *Z*‐value: 4.44, *p* < 0.05, and Bray–Curtis *Z*‐value: 3.66, *p* < 0.05), whereas distance was only significant, and positively correlated, with the Bray–Curtis index (*Z*‐value: 4.39, *p* < 0.05) (Figure 4b) (Table S3).

All models underwent residual diagnostics for hierarchical regression models (DHARMa) and VIF (<3) diagnostic analysis (Figure S3; Table S6). The stratified block bootstrap per year indicated consistent and statistic robust effects.

**FIGURE 5:**
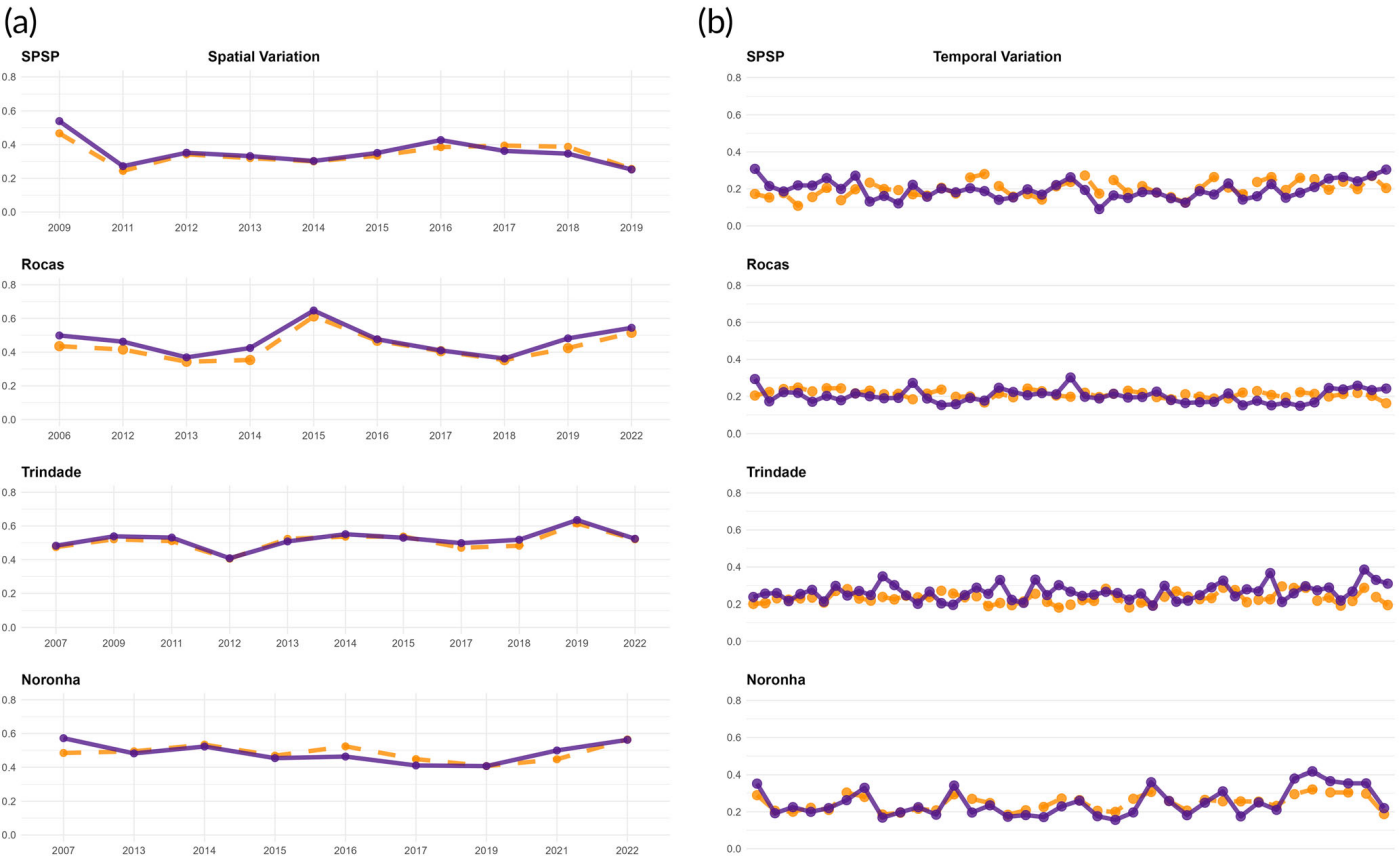
(a) Spatial and (b) temporal dissimilarity variation in the islands; purple continuous lines represent the Bray–Curtis dissimilarity and orange dotted lines Sørensen dissimilarity. *Y*‐axis represents the dissimilarity, and *X‐*axis represents each result of the 999 permutations for each island.

**FIGURE 6:**
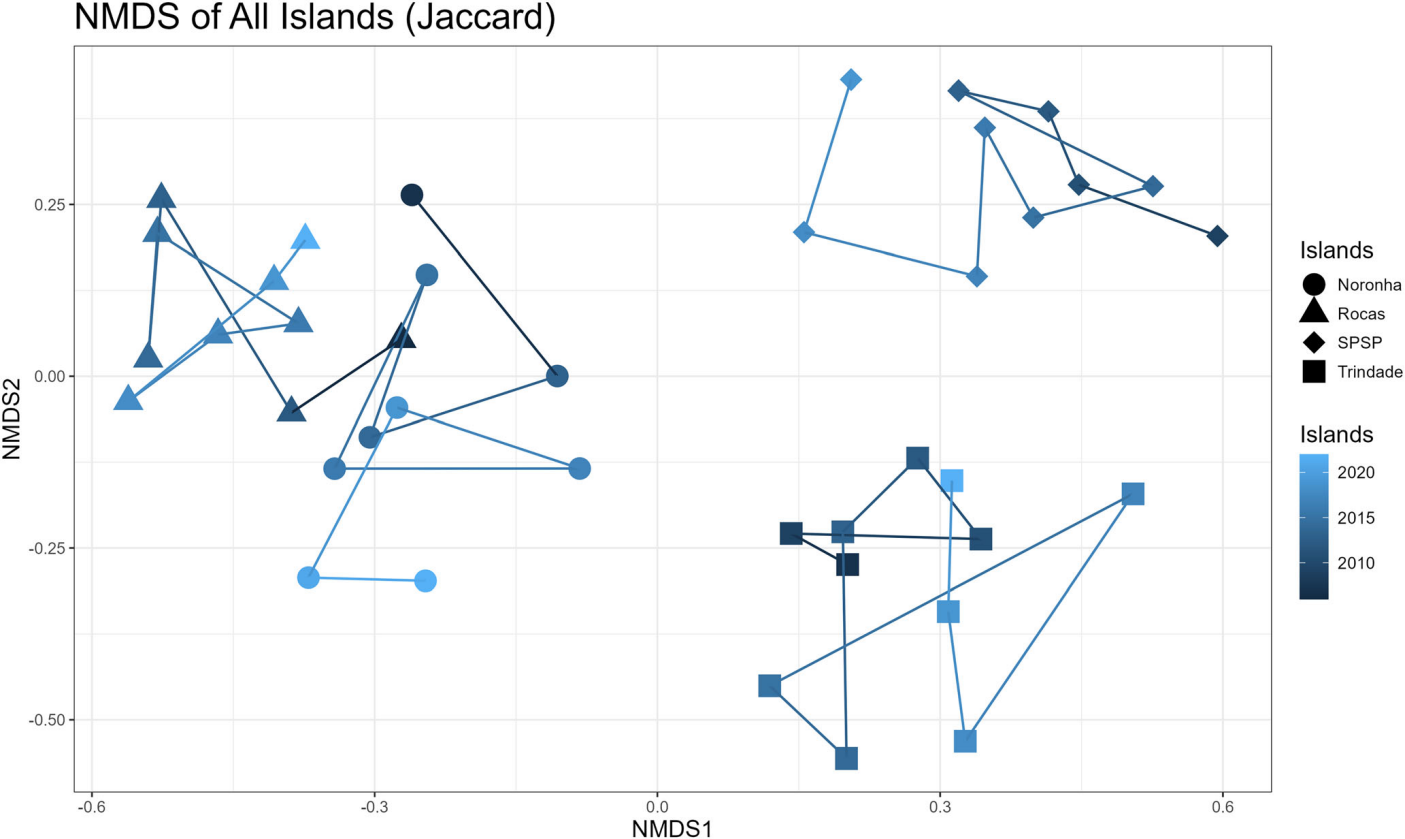
Non‐metric multidimensional scaling (nMDS) ordination of all the islands, showing the variation along the years, using the Jaccard index for the presence and absence matrix of the species.

Additionally, Sørensen and Bray–Curtis indices, despite being analogous metrics, exhibited distinct spatial and temporal patterns. Both indices were relatively consistent regarding the spatial variation, although some islands presented spikes of dissimilarity in some years [e.g. Rocas 2015, Sørensen = 0.61, 95% confidence interval (CI) [0.388, 0.483], Bray–Curtis = 0.64 95% CI [0.420, 0.519]; SPSP 2009, Sørensen = 0.46, 95% CI [0.303, 0.385], Bray–Curtis = 0.53, 95% CI [0.310, 0.404]] (Figure 5). Temporally, both indices exhibited distinct trends yet remained relatively similar in magnitude (Figure 5). Although changing temporarily, the assemblages exhibited no directional changes through time (Figure 6).

Significant temporal differences in assemblage composition were obtained for all islands (PERMANOVA and multivariate dispersion tests; Table S4). Dispersion heterogeneity was detected for Rocas (Bray–Curtis, *F* = 4.5; Jaccard, *F* = 3.56) and SPSP (Bray–Curtis, *F* = 8.43; Jaccard, *F* = 6.71), indicating that temporal dissimilarities in these sites may partially reflect differences in within‐year variability.

## 4 DISCUSSION

Our study provides novel empirical support for the TIB (MacArthur & Wilson, 1963, 1967) by demonstrating how island area and isolation contribute to the richness and stability of reef fish assemblages in the southwestern Atlantic oceanic islands. Island size contributed most to species richness, which is consistent with regional‐scale findings based on historical checklists (Hachich et al., 2015). Isolation and distance to the continents have less effect on species richness, mainly due to the high dispersal potential of fishes (Hachich et al., 2015; Mazzei et al., 2021), which accumulate with time. Nevertheless, according to the TIB predictions and species–area relationship (Lewinsohn & Ré Jorge, 2024; MacArthur & Wilson, 1967), larger islands support more species due to greater habitat availability, increased heterogeneity and proportionally lower extinction rates (García‐Charton & Pérez‐Ruzafa, 2001; Komyakova et al., 2013). Therefore, the match between small‐scale, species pool and checklist richness suggests the influence of regional enrichment (Bellwood & Hughes, 2001; Pinheiro et al., 2023) or species packing (Barneche et al., 2019) processes, sustaining the TIB in multiple scales.

Spatial dissimilarity in the reef fish assemblage also followed the premises of TIB: larger islands exhibited higher assemblage dissimilarity in both the Sørensen and Bray–Curtis indices, exhibiting similar spatial trends. This concordant pattern is consistent with their theoretical analogies (Baselga, 2013, 2017). Particularly, the turnover component (Simpson component) and its counterpart abundance balance likely reflect the diversity of microhabitats and species sorting processes (Koivunen et al., 2025; Magurran et al., 2019). Therefore, the higher richness found in larger islands is based on the diversity of species distribution patterns, where different species are found in distinct places of the island.

Despite their conceptual similarities (Baselga, 2013, 2017), the trajectories of the Sørensen and Bray–Curtis indices diverged through time. Turnover remained the primary driver of Sørensen dissimilarity, supporting the notion that species replacement is the central mechanism structuring reef fish assemblages across different spatial and temporal scales, from local to provincial (Maxwell et al., 2022) and from ecological to evolutionary levels (Cowman & Belwood, 2012; Pinheiro et al., 2017). The Bray–Curtis dissimilarity, on the contrary, showed a greater contribution of the abundance gradient component and, together with the balance component, explained the variation observed in the largest islands. This result indicates that large islands are more susceptible to abundance shifts, as a result of competition or predator–prey species, but also as influenced by recruitment pulses or sharp population declines. Therefore, differences may reflect internal population dynamics (such as abundance shifts based on species interactions and population expansion and contraction), in addition to species replacement, altogether contributing to assemblage maintenance over time. These results indicate that the dynamic equilibrium is shaped by both colonization–extinction cycles and internal community dynamics that can be perceived in ecological scales.

The temporal stability, however, did not follow the initial prediction based on coral reef ecosystems. Smaller and more isolated islands exhibited lower dissimilarity, which could be related to high levels of endemism of the oceanic islands (Floeter et al., 2007; Pinheiro et al., 2018, 2020). This result suggests that endemic species and evolutionary processes, such as selection and adaptation, may confer resilience to the local assemblage structure over time, excluding common and abundant species through species losses, which could lead to different results of β diversity (Tatsumi et al., 2020), thereby shaping the dynamics of those insular systems (Fernández‐Cisternas et al., 2021). Isolated populations are subjected to strong environmental filtering (Mazzei et al., 2021; Pinheiro et al., 2018), which might result in the establishment of competitive assemblages, especially in small islands. Conversely, large islands exhibited more dynamic assemblage changes over time, exhibiting contributions of balances related to greater species richness and increased habitat heterogeneity, but also opportunities for colonization and species turnover.

Interestingly, distance from the mainland and isolation exhibited distinct temporal Bray–Curtis trends. This is because Trindade island, the most isolated and larger, is also strongly connected (Pinheiro et al., 2017). It is close to the Martin Vaz Archipelago and part of a seamount chain. Distance, therefore, does not necessarily reflect remoteness, which could lead to misconception and divergent patterns as shown here. A growing body of research is using different metrics to estimate isolation instead of distance (Hachich et al., 2015; Maxwell et al., 2022; Quimbayo et al., 2019).

The natural temporal balance of species observed in this study may explain the recent surge in the biodiversity records in the southwestern Atlantic oceanic islands, aligning with discoveries made during expeditions over the past 20 years (Carvalho, 1950; Feitoza et al., 2003; Lubbock & Edwards, 1981; Murray, 1902; Pinheiro et al., 2009, 2015, 2020). Our results suggest that even over a short ecological time frame, reef fish assemblage can exhibit detectable ecological dynamics linked to the TIB principles. Cyclic shifts in composition underscore the importance of long‐term monitoring to capture the temporal dimensions of island assemblage (Maxwell et al., 2022; Wilcox et al., 2017). For instance, the assemblages of smaller islands are poorer and exhibit less temporal variation. Lower functional redundancy in these smaller islands still needs to be understood in terms of the temporal influence on community structure resilience. Such a scenario reflects food webs that could be more susceptible to anthropogenic impacts, and therefore, those on the top list as priorities for conservation initiatives.

The temporal and spatial variation in marine organisms in oceanic islands remains understudied (Dawson, 2015; Lamy et al., 2015; Quimbayo et al., 2019) despite their significance for ecological and evolutionary research. This study reinforces the foundational predictions of island biogeography theory while revealing that long‐term ecological processes (particularly those related to abundance and species turnover) can operate on a short time scale. Although similar patterns were observed spatially and temporally, there are nuances that vary between those diversity analyses, highlighting the need to integrate temporal dimensions into biogeographic frameworks. Smaller and more isolated islands present lower species richness and functional redundancy but exhibited more stable assemblages through time. Larger and more connected islands, despite being richer, exhibit greater internal population and species changes ruling their assemblages. These findings highlight the importance of sustained ecological monitoring in oceanic islands. Long‐term datasets are essential to disentangling ecological stability, enhancing our understanding of assemblage resilience and guiding conservation strategies in isolated marine ecosystems.

## AUTHOR CONTRIBUTIONS

Esteban Jorcin Nogueira: conceptualization, data curation, formal analysis, methodology, project administration, visualization, investigation, writing – original draft and editing. Camille Mellin: writing – original draft, methodology, visualization, investigation and editing. Carlos E. L. Ferreira: data curation, funding acquisition, investigation, writing – original draft and editing. Hudson Tercio Pinheiro: conceptualization, data curation, funding acquisition, project administration, supervision, writing – original draft and editing.

## FUNDING INFORMATION

Fieldwork funding was provided by CNPq through the grants Long Term Ecological Research of Reef Communities at Oceanic Islands – PELD ILOC (CNPq 441327/2020‐6; principal investigator: Carlos E. L. Ferreira) and SISBIOTA–Mar. Additionally, Esteban Jorcin Nogueira and Hudson Tercio Pinheiro received fellowships and funding from the Fundação de Amparo à Pesquisa do Estado de São Paulo (FAPESP 2023/12551‐3, 2021/07039‐6 and 2019/24215‐2). Carlos E. L. Ferreira received productivity grants from Conselho Nacional de Desenvolvimento Científico e Tecnológico (CNPq‐Brazil, 310219/2023‐0).

## Supporting information


**Data S1.** Supplementary Material.
**Table SM1.** Species list for each island.
**Table SM2.** Kruskal‐Wallis test Søorensen and Bray‐Curtis and its components (Temporal and Spatial), as well for Species pool and Transect Richness.
**Table SM3.** Temporal and Spatial Sørensen and Bray‐Curtis GLMMs (Significance codes: *p* < 0 ‘***’, *p* < 0.001 ‘**’, *p* < 0.01 ‘**’, *p* < 0.05 ‘.’).
**Table SM4.** Permutational Multivariate Analysis of Variance and Multivariate homogeneity of groups dispersions (betadisper) for reef fish assemblages among years within each island. (Significance codes: *p* < 0 ‘***’, *p* < 0.001 ‘**’, *p* < 0.01 ‘**’, *p* < 0.05 ‘.’).
**Table SM5.** Mean values of Beta diversity and its components per island and year.
**Table SM6.** Variance‐inflation factors (VIF) values.
**Table SM7.** Temporal and Spatial Sørensen and Bray‐Curtis GLMMs using Logit transform with Gaussian (Significance codes: *p* < 0 ‘***’, *p* < 0.001 ‘**’, *p* < 0.01 ‘**’, *p* < 0.05 ‘.’).
**Table SM8.** Kruskal‐Wallis test Sørensen and Bray‐Curtis and its components (Temporal and Spatial), as well for Species pool and Transect Richness, using Hellinger transformed data.
**Figure SM1.** Rarefaction curves for all Islands.
**Figure SM2.** Heatmaps of the Abundance of Species (30 most abundant) in all islands over the years.
**Figure SM3.** DHARMa residuals for the GLMMs (Temporal and Spatial, Sørensen and Bray‐Curtis).
