# Supplementary material for: The theory of island biogeography and the stability of oceanic reef fish communities
Source: J Fish Biol. 2026 Feb 22;108(6):2041–52. doi: 10.1111/jfb.70367 (PMC13357251; doi:10.1111/jfb.70367)
Supplement: Supplementary file 1 — Data S1. Supplementary Material. Table SM1. Species list for each island. Table SM2. Kruskal‐Wallis test Søorensen and Bray‐Curtis and its components (Temporal and Spatial), as well for Species pool and Transect Richness. Table SM3. Temporal and Spatial Sørensen and Bray‐Curtis GLMMs (Significance codes: p < 0 ‘***’, p < 0.001 ‘**’, p < 0.01 ‘**’, p < 0.05 ‘.’). Table SM4. Permutational Multivariate Analysis of Variance and Multivariate homogeneity of groups dispersions (betadisper) for reef fish assemblages among years within each island. (Significance codes: p < 0 ‘***’, p < 0.001 ‘**’, p < 0.01 ‘**’, p < 0.05 ‘.’). Table SM5. Mean values of Beta diversity and its components per island and year. Table SM6. Variance‐inflation factors (VIF) values. Table SM7. Temporal and Spatial Sørensen and Bray‐Curtis GLMMs using Logit transform with Gaussian (Significance codes: p < 0 ‘***’, p < 0.001 ‘**’, p < 0.01 ‘**’, p < 0.05 ‘.’). Table SM8. Kruskal‐Wallis test Sørensen and Bray‐Curtis and its components (Temporal and Spatial), as well for Species pool and Transect Richness, using Hellinger transformed data. Figure SM1. Rarefaction curves for all Islands. Figure SM2. Heatmaps of the Abundance of Species (30 most abundant) in all islands over the years. Figure SM3. DHARMa residuals for the GLMMs (Temporal and Spatial, Sørensen and Bray‐Curtis). [file JFB-108-2041-s001.docx]

**Supplementary material**

**The theory of island biogeography and the stability of oceanic reef fish communities**

**Esteban Jorcin Nogueira¹ - CEBIMar - USP**

**Carlos E. L. Ferreira² - UFF**

**Camille Mellin³ - University of Adelaide**

**Hudson Tercio Pinheiro¹ - CEBIMar - USP**

Table SM1- Species list for each island

| **Family** | **Species** | **Noronha** | **Rocas** | **SPSP** | **Trindade** |
| --- | --- | --- | --- | --- | --- |
| *Acanthuridae* | *Acanthurus bahianus* | X | X |  | X |
|  | *Acanthurus chirurgus* | X | X |  |  |
|  | *Acanthurus coeruleus* | X | X |  | X |
| *Albuliformes* | *Albula vulpes* | X | X |  |  |
| *Apogonidae* | *Apogon americanus* |  | X | X | X |
|  | *Phaeoptyx pigmentaria* |  | X |  |  |
| *Aulosomidae* | *Aulostomus strigosus* | X |  | X |  |
| *Balistidae* | *Balistes vetula* |  | X |  | X |
|  | *Canthidermis sufflamen* | X | X | X | X |
|  | *Melichthys niger* | X | X | X | X |
| *Belonidae* | *Belone belone* | X |  |  |  |
|  | *Platybelone argalus* |  | X |  | X |
|  | *Strongylura timucu* |  | X |  |  |
| *Blenniidae* | *Entomacrodus vomerinus* |  |  |  | X |
|  | *Hypleurochilus brasil* |  |  |  | X |
|  | *Ophioblennius atlanticus* | X | X |  | X |
|  | *Ophioblennius trinitatis* | X | X | X | X |
|  | *Parablennius marmoreus* |  |  |  | X |
| *Carangidae* | *Carangoides bartholomaei* | X | X | X |  |
|  | *Caranx bartholomaei* | X |  |  |  |
|  | *Caranx crysos* | X | X | X | X |
|  | *Caranx latus* | X | X | X | X |
|  | *Caranx lugubris* | X | X | X | X |
|  | *Caranx ruber* | X | X | X | X |
|  | *Elagatis bipinnulata* |  |  |  | X |
|  | *Selar crumenophthalmus* |  |  |  | X |
|  | *Seriola dumerili* | X |  |  |  |
|  | *Seriola rivoliana* |  | X | X | X |
| *Carcharhinidae* | *Carcharhinus perezi* | X |  |  |  |
| *Chaenopsidae* | *Emblemariopsis signifera* | X |  | X |  |
|  | *Epinephelus adscensionis* |  |  |  | X |
| *Chaetodontidae* | *Chaetodon ocellatus* | X | X |  | X |
|  | *Chaetodon striatus* |  |  | X | X |
|  | *Prognathodes brasiliensis* | X |  |  | X |
|  | *Prognathodes obliquus* |  |  | X |  |
| *Cirrhitidae* | *Amblycirrhitus pinos* | X |  |  | X |
| *Dactylopteridae* | *Dactylopterus volitans* |  |  | X | X |
| *Dasyatidae* | *Hypanus americanus* | X |  |  |  |
| *Diodontidae* | *Chilomycterus reticulatus* |  |  | X | X |
|  | *Chilomycterus spinosus* |  |  |  | X |
|  | *Diodon holocanthus* |  |  | X | X |
|  | *Diodon hystrix* | X | X |  | X |
| *Echeneidae* | *Echeneis naucrates* |  | X |  |  |
| *Epinephelidae* | *Cephalopholis fulva* | X | X |  | X |
|  | *Dermatolepis inermis* | X | X |  | X |
| *Fistulariidae* | *Fistularia petimba* |  |  |  | X |
|  | *Fistularia tabacaria* | X |  |  |  |
| *Ginglymostomatidae* | *Ginglymostoma cirratum* | X | X |  |  |
| *Gobiidae* | *Coryphopterus* spp | X | X |  | X |
|  | *Ctenogobius saepepallens* |  | X |  |  |
|  | *Elacatinus phthirophagus* | X | X |  |  |
|  | *Elacatinus pridisi* |  |  |  | X |
|  | *Gnatholepis thompsoni* | X | X |  | X |
| *Grammistidae* | *Rypticus saponaceus* | X | X | X | X |
| *Haemulidae* | *Anisotremus surinamensis* | X | X |  | X |
|  | *Anisotremus virginicus* | X |  |  |  |
|  | *Haemulon chrysargyreum* | X | X |  | X |
|  | *Haemulon parra* | X | X |  |  |
|  | *Orthopristis ruber* |  | X |  |  |
| *Hemiramphidae* | *Hemiramphus brasiliensis* |  | X |  | X |
| *Holocentridae* | *Holocentrus adscensionis* | X | X | X | X |
|  | *Sargocentron bullisi* |  |  |  | X |
| *Hybrid* | *Menephorus punticulatus* | X |  |  | X |
| *Kyphosidae* | *Kyphosus* spp | X | X | X | X |
| *Labridae* | *Bodianus insularis* |  |  | X |  |
|  | *Bodianus pulchellus* | X |  |  | X |
|  | *Bodianus rufus* |  |  |  | X |
|  | *Bothus lunatus* | X | X |  | X |
|  | *Clepticus brasiliensis* | X |  | X | X |
|  | *Doratonotus megalepis* | X | X |  | X |
|  | *Halichoeres brasiliensis* |  |  |  | X |
|  | *Halichoeres dimidiatus* | X |  |  |  |
|  | *Halichoeres penrosei* |  |  |  | X |
|  | *Halichoeres poeyi* |  |  |  | X |
|  | *Halichoeres rubrovirens* |  |  |  | X |
|  | *Thalassoma noronhanum* | X | X | X | X |
|  | *Xyrichtys novacula* |  |  |  | X |
| *Labrisomidae* | *Gobioclinus kalisherae* | X | X |  |  |
|  | *Labrisomus nuchipinnis* | X |  |  | X |
|  | *Malacoctenus brunoi* |  |  |  | X |
|  | *Malacoctenus lianae* | X | X | X |  |
| *Lutjanidae* | *Lutjanus jocu* | X | X | X |  |
|  | *Ocyurus chrysurus* |  | X |  |  |
| *Malacanthidae* | *Malacanthus plumieri* | X | X |  | X |
| *Monacanthidae* | *Aluterus monoceros* | X |  |  |  |
|  | *Aluterus scriptus* | X | X | X | X |
|  | *Cantherhines macrocerus* | X | X | X | X |
|  | *Cantherhines pullus* | X | X | X | X |
| *Mullidae* | *Mulloidichthys martinicus* | X | X |  | X |
|  | *Pseudupeneus maculatus* | X | X |  | X |
| *Muraenidae* | *Echidna catenata* | X |  |  | X |
|  | *Enchelycore anatina* |  |  | X |  |
|  | *Enchelycore carychroa* |  | X |  | X |
|  | *Enchelycore nigricans* |  | X | X | X |
|  | *Gymnothorax funebris* | X |  | X |  |
|  | *Gymnothorax miliaris* | X | X | X | X |
|  | *Gymnothorax moringa* | X |  |  | X |
|  | *Gymnothorax vicinus* |  | X |  |  |
|  | *Muraena melanotis* |  |  | X |  |
|  | *Muraena pavonina* | X | X | X | X |
| *Ophichthidae* | *Myrichthys breviceps* |  |  |  | X |
|  | *Myripristis jacobus* | X | X | X | X |
|  | *Ophichthus ophis* |  |  |  | X |
| *Opistognathidae* | *Opistognathus aurifrons* |  |  |  | X |
| *Ostraciidae* | *Acanthostracion polygonius* | X | X |  | X |
|  | *Acanthostracion quadricornis* | X |  |  | X |
| *Ostraciidae* | *Lactophrys trigonus* | X | X |  |  |
| *Pempheridae* | *Pempheris schomburgkii* | X | X |  | X |
| *Pomacanthidae* | *Centropyge aurantonotus* | X |  |  |  |
|  | *Holacanthus ciliaris* | X |  | X |  |
|  | *Holacanthus tricolor* |  |  |  | X |
|  | *Pomacanthus paru* | X | X | X |  |
|  | *Abudefduf saxatilis* | X | X | X | X |
| *Pomacentridae* | *Chromis jubauna* |  |  |  | X |
|  | *Chromis multilineata* | X | X | X | X |
|  | *Microspathodon chrysurus* |  |  |  | X |
|  | *Stegastes pictus* | X | X |  | X |
|  | *Stegastes rocasensis* | X | X | X |  |
|  | *Stegastes sanctipauli* |  |  | X |  |
|  | *Stegastes trindadensis* |  |  |  | X |
| *Priacanthidae* | *Heteropriacanthus cruentatus* | X |  |  | X |
|  | *Priacanthus arenatus* | X |  |  |  |
| *Scaridae* | *Nicholsina usta* |  | X |  |  |
|  | *Scarus trispinosus* |  | X |  |  |
|  | *Sparisoma amplum* | X | X | X | X |
|  | *Sparisoma axillare* | X | X |  | X |
|  | *Sparisoma frondosum* | X | X | X | X |
|  | *Sparisoma radians* | X | X |  |  |
|  | *Sparisoma rocha* |  |  |  | X |
| *Scorpaenidae* | *Scorpaena brasiliensis* |  |  | X |  |
|  | *Scorpaena plumieri* |  |  |  | X |
| *Serranidae* | *Mycteroperca interstitialis* |  |  |  | X |
|  | *Mycteroperca venenosa* |  |  |  | X |
|  | *Paranthias furcifer* | X | X |  | X |
| *Sparidae* | *Diplodus argenteus* |  |  |  | X |
| *Sphyraenidae* | *Sphyraena barracuda* | X | X | X | X |
|  | *Sphyraena borealis* |  | X |  |  |
|  | *Sphyraena picudilla* | X |  |  |  |
| *Synodontidae* | *Synodus synodus* | X | X |  | X |
| *Tetraodontidae* | *Canthigaster figueiredoi* | X | X |  | X |
|  | *Sphoeroides spengleri* |  |  |  | X |
| *Trachinotinae* | *Trachinotus falcatus* | X |  |  |  |
| *Tripterygiinae* | *Enneanectes altivelis* |  | X |  |  |
|  | *Enneanectes smithi* |  |  | X |  |

Table SM2- Kruskal-Wallis test Sørensen and Bray-Curtis and its components (Temporal and Spatial), as well for Species pool and Transect Richness

| Temporal Dissimilarity | | | | | | | | | |
| --- | --- | --- | --- | --- | --- | --- | --- | --- | --- |
| Metric | group1 | group2 | n1 | n2 | statistic | p | p.adj | p.adj.signif | p.adj.FDR.global |
| Bray-Curtis | Noronha | Rocas | 36 | 45 | -3,03533033 | 0,00240272 | 0,00240272 | ** | 0,00864981 |
| Bray-Curtis | Noronha | SPSP | 36 | 45 | -3,35968058 | 0,00078033 | 0,00078033 | *** | 0,00312131 |
| Bray-Curtis | Noronha | Trindade | 36 | 55 | 2,2915766 | 0,02193009 | 0,02193009 | * | 0,05263221 |
| Bray-Curtis | Rocas | SPSP | 45 | 45 | -0,34402539 | 0,73082719 | 0,73082719 | ns | 0,77381703 |
| Bray-Curtis | Rocas | Trindade | 45 | 55 | 5,8206418 | 5,8622E-09 | 5,8622E-09 | **** | 1,0552E-07 |
| Bray-Curtis | SPSP | Trindade | 45 | 55 | 6,18145868 | 6,3512E-10 | 6,3512E-10 | **** | 2,2864E-08 |
| Balanced | Noronha | Rocas | 36 | 45 | -0,01896787 | 0,98486674 | 0,98486674 | ns | 0,98486674 |
| Balanced | Noronha | SPSP | 36 | 45 | -1,95084543 | 0,05107544 | 0,05107544 | ns | 0,09193578 |
| Balanced | Noronha | Trindade | 36 | 55 | 0,47652611 | 0,63369961 | 0,63369961 | ns | 0,71291206 |
| Balanced | Rocas | SPSP | 45 | 45 | -2,04906559 | 0,0404557 | 0,0404557 | * | 0,07721368 |
| Balanced | Rocas | Trindade | 45 | 55 | 0,52933289 | 0,59657454 | 0,59657454 | ns | 0,70112826 |
| Balanced | SPSP | Trindade | 45 | 55 | 2,67841101 | 0,00739724 | 0,00739724 | ** | 0,01902147 |
| Gradient | Noronha | Rocas | 36 | 45 | -1,79672967 | 0,07237855 | 0,07237855 | ns | 0,11987667 |
| Gradient | Noronha | SPSP | 36 | 45 | -0,96024745 | 0,33693069 | 0,33693069 | ns | 0,44924092 |
| Gradient | Noronha | Trindade | 36 | 55 | 1,79122019 | 0,07325797 | 0,07325797 | ns | 0,11987667 |
| Gradient | Rocas | SPSP | 45 | 45 | 0,88722338 | 0,37495865 | 0,37495865 | ns | 0,48208969 |
| Gradient | Rocas | Trindade | 45 | 55 | 3,9091372 | 9,2626E-05 | 9,2626E-05 | **** | 0,00047636 |
| Gradient | SPSP | Trindade | 45 | 55 | 2,97860948 | 0,0028956 | 0,0028956 | ** | 0,00868679 |
| Sørensen | Noronha | Rocas | 36 | 45 | -3,63756143 | 0,00027523 | 0,00027523 | *** | 0,00123854 |
| Sørensen | Noronha | SPSP | 36 | 45 | -4,75856199 | 1,9498E-06 | 1,9498E-06 | **** | 1,4038E-05 |
| Sørensen | Noronha | Trindade | 36 | 55 | -1,09536203 | 0,27335806 | 0,27335806 | ns | 0,37849578 |
| Sørensen | Rocas | SPSP | 45 | 45 | -1,18900064 | 0,23443942 | 0,23443942 | ns | 0,33759276 |
| Sørensen | Rocas | Trindade | 45 | 55 | 2,87828811 | 0,0039984 | 0,0039984 | ** | 0,01107249 |
| Sørensen | SPSP | Trindade | 45 | 55 | 4,1253225 | 3,7022E-05 | 3,7022E-05 | **** | 0,00022213 |
| Simpson | Noronha | Rocas | 36 | 45 | -2,07935065 | 0,03758513 | 0,03758513 | * | 0,07721368 |
| Simpson | Noronha | SPSP | 36 | 45 | -4,91314756 | 8,9626E-07 | 8,9626E-07 | **** | 8,1144E-06 |
| Simpson | Noronha | Trindade | 36 | 55 | -0,51901636 | 0,60374933 | 0,60374933 | ns | 0,70112826 |
| Simpson | Rocas | SPSP | 45 | 45 | -3,00569552 | 0,00264974 | 0,00264974 | ** | 0,00867188 |
| Simpson | Rocas | Trindade | 45 | 55 | 1,7595817 | 0,07847876 | 0,07847876 | ns | 0,12283631 |
| Simpson | SPSP | Trindade | 45 | 55 | 4,91198176 | 9,016E-07 | 9,016E-07 | **** | 8,1144E-06 |
| Nestedness | Noronha | Rocas | 36 | 45 | -2,12961761 | 0,0332032 | 0,0332032 | * | 0,07470719 |
| Nestedness | Noronha | SPSP | 36 | 45 | -0,20058523 | 0,84102291 | 0,84102291 | ns | 0,86505214 |
| Nestedness | Noronha | Trindade | 36 | 55 | -0,60759552 | 0,54345577 | 0,54345577 | ns | 0,67463475 |
| Nestedness | Rocas | SPSP | 45 | 45 | 2,04604782 | 0,04075167 | 0,04075167 | * | 0,07721368 |
| Nestedness | Rocas | Trindade | 45 | 55 | 1,72102726 | 0,08524588 | 0,08524588 | ns | 0,12786882 |
| Nestedness | SPSP | Trindade | 45 | 55 | -0,4248858 | 0,67091993 | 0,67091993 | ns | 0,73191265 |
| Spatial Dissimilarity | | | | | | | | | |
| Metric | group1 | group2 | n1 | n2 | statistic | p | p.adj | p.adj.signif | p.adj.FDR.global |
| Bray-Curtis | Noronha | Rocas | 2277 | 2530 | -7,87528723 | 3,3996E-15 | 3,3996E-15 | **** | 5,32113E-15 |
| Bray-Curtis | Noronha | SPSP | 2277 | 2530 | -31,7020066 | 1,458E-220 | 1,458E-220 | **** | 6,5602E-220 |
| Bray-Curtis | Noronha | Trindade | 2277 | 2783 | 8,00771048 | 1,1686E-15 | 1,1686E-15 | **** | 2,00338E-15 |
| Bray-Curtis | Rocas | SPSP | 2530 | 2530 | -24,4796271 | 2,435E-132 | 2,435E-132 | **** | 8,7653E-132 |
| Bray-Curtis | Rocas | Trindade | 2530 | 2783 | 16,5189368 | 2,6808E-61 | 2,6808E-61 | **** | 6,03185E-61 |
| Bray-Curtis | SPSP | Trindade | 2530 | 2783 | 41,5746339 | 0 | 0 | **** | 0 |
| Balanced | Noronha | Rocas | 2277 | 2530 | -16,3516665 | 4,2324E-60 | 4,2324E-60 | **** | 8,96283E-60 |
| Balanced | Noronha | SPSP | 2277 | 2530 | -33,322217 | 1,841E-243 | 1,841E-243 | **** | 1,3254E-242 |
| Balanced | Noronha | Trindade | 2277 | 2783 | 2,33943171 | 0,0193131 | 0,0193131 | * | 0,02172724 |
| Balanced | Rocas | SPSP | 2530 | 2530 | -17,4355832 | 4,4302E-68 | 4,4302E-68 | **** | 1,13919E-67 |
| Balanced | Rocas | Trindade | 2530 | 2783 | 19,6016301 | 1,4975E-85 | 1,4975E-85 | **** | 4,1469E-85 |
| Balanced | SPSP | Trindade | 2530 | 2783 | 37,4475184 | 6,592E-307 | 6,592E-307 | **** | 7,9104E-306 |
| Gradient | Noronha | Rocas | 2277 | 2530 | 7,07151111 | 1,5326E-12 | 1,5326E-12 | **** | 2,20688E-12 |
| Gradient | Noronha | SPSP | 2277 | 2530 | 2,91709822 | 0,00353304 | 0,00353304 | ** | 0,004102891 |
| Gradient | Noronha | Trindade | 2277 | 2783 | 7,94647862 | 1,9189E-15 | 1,9189E-15 | **** | 3,13999E-15 |
| Gradient | Rocas | SPSP | 2530 | 2530 | -4,26825349 | 1,9701E-05 | 1,9701E-05 | **** | 2,44563E-05 |
| Gradient | Rocas | Trindade | 2530 | 2783 | 0,7381968 | 0,46039487 | 0,46039487 | ns | 0,473549004 |
| Gradient | SPSP | Trindade | 2530 | 2783 | 5,10689353 | 3,275E-07 | 3,275E-07 | **** | 4,36664E-07 |
| Sørensen | Noronha | Rocas | 2277 | 2530 | -15,8607209 | 1,1853E-56 | 1,1853E-56 | **** | 2,37051E-56 |
| Sørensen | Noronha | SPSP | 2277 | 2530 | -32,8270283 | 2,424E-236 | 2,424E-236 | **** | 1,2464E-235 |
| Sørensen | Noronha | Trindade | 2277 | 2783 | 4,5607862 | 5,0962E-06 | 5,0962E-06 | **** | 6,55232E-06 |
| Sørensen | Rocas | SPSP | 2530 | 2530 | -17,4312239 | 4,7812E-68 | 4,7812E-68 | **** | 1,14748E-67 |
| Sørensen | Rocas | Trindade | 2530 | 2783 | 21,3704445 | 2,517E-101 | 2,517E-101 | **** | 7,5517E-101 |
| Sørensen | SPSP | Trindade | 2530 | 2783 | 39,211871 | 0 | 0 | **** | 0 |
| Simpson | Noronha | Rocas | 2277 | 2530 | -24,0483501 | 8,686E-128 | 8,686E-128 | **** | 2,8428E-127 |
| Simpson | Noronha | SPSP | 2277 | 2530 | -32,8624053 | 7,574E-237 | 7,574E-237 | **** | 4,5447E-236 |
| Simpson | Noronha | Trindade | 2277 | 2783 | 0,103381 | 0,91766059 | 0,91766059 | ns | 0,917660593 |
| Simpson | Rocas | SPSP | 2530 | 2530 | -9,05558092 | 1,3584E-19 | 1,3584E-19 | **** | 2,44517E-19 |
| Simpson | Rocas | Trindade | 2530 | 2783 | 25,3951064 | 2,856E-142 | 2,856E-142 | **** | 1,1425E-141 |
| Simpson | SPSP | Trindade | 2530 | 2783 | 34,663789 | 2,769E-263 | 2,769E-263 | **** | 2,492E-262 |
| Nestedness | Noronha | Rocas | 2277 | 2530 | 9,51515681 | 1,8144E-21 | 1,8144E-21 | **** | 3,43779E-21 |
| Nestedness | Noronha | SPSP | 2277 | 2530 | 6,29659635 | 3,0425E-10 | 3,0425E-10 | **** | 4,21272E-10 |
| Nestedness | Noronha | Trindade | 2277 | 2783 | 7,51483872 | 5,6981E-14 | 5,6981E-14 | **** | 8,54717E-14 |
| Nestedness | Rocas | SPSP | 2530 | 2530 | -3,30675653 | 0,00094383 | 0,00094383 | *** | 0,001132595 |
| Nestedness | Rocas | Trindade | 2530 | 2783 | -2,2755149 | 0,02287506 | 0,02287506 | * | 0,024954611 |
| Nestedness | SPSP | Trindade | 2530 | 2783 | 1,10905831 | 0,26740503 | 0,26740503 | ns | 0,283134736 |
| Spatial Richness | | | | | | | | | |
| Metric | group1 | group2 | n1 | n2 | statistic | p | p.adj | p.adj.signif | p.adj.FDR.global |
| Richness | Noronha | Rocas | 918 | 822 | -0,29208111 | 0,77022461 | 0,77022461 | ns | 0,92426953 |
| Richness | Noronha | SPSP | 918 | 506 | -5,89368213 | 3,7768E-09 | 3,7768E-09 | **** | 1,1331E-08 |
| Richness | Noronha | Trindade | 918 | 1530 | -0,37741863 | 0,70586253 | 0,70586253 | ns | 0,92426953 |
| Richness | Rocas | SPSP | 822 | 506 | -5,52685193 | 3,2603E-08 | 3,2603E-08 | **** | 6,5206E-08 |
| Richness | Rocas | Trindade | 822 | 1530 | -0,04002765 | 0,96807108 | 0,96807108 | ns | 0,96807108 |
| Richness | SPSP | Trindade | 506 | 1530 | 6,05597217 | 1,3957E-09 | 1,3957E-09 | **** | 8,3743E-09 |
| Temporal Dissimilarity | | | | | | | | | |
| Metric | group1 | group2 | n1 | n2 | statistic | p | p.adj | p.adj.signif | p.adj.FDR.global |
| Richness | Noronha | Rocas | 9 | 10 | -0,20892577 | 0,83450618 | 0,83450618 | ns | 0,95883691 |
| Richness | Noronha | SPSP | 9 | 10 | -0,89776025 | 0,36931338 | 0,36931338 | ns | 0,95825054 |
| Richness | Noronha | Trindade | 9 | 11 | -0,16340112 | 0,87020262 | 0,87020262 | ns | 0,95883691 |
| Richness | Rocas | SPSP | 10 | 10 | -0,70771015 | 0,47912527 | 0,47912527 | ns | 0,95825054 |
| Richness | Rocas | Trindade | 10 | 11 | 0,05161319 | 0,95883691 | 0,95883691 | ns | 0,95883691 |
| Richness | SPSP | Trindade | 10 | 11 | 0,77597762 | 0,4377622 | 0,4377622 | ns | 0,95825054 |


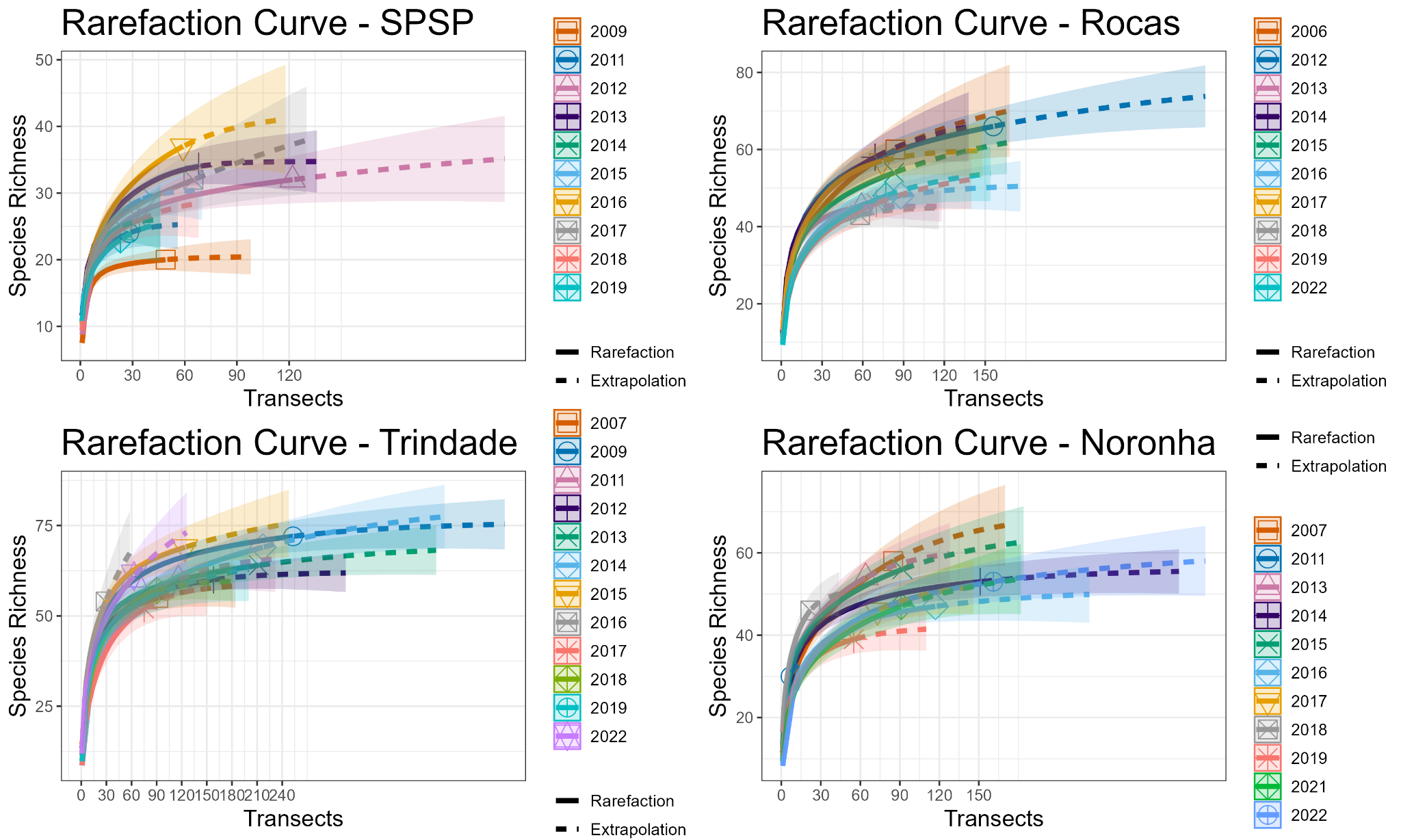


Figure SM 1 - Rarefaction curves for all Islands


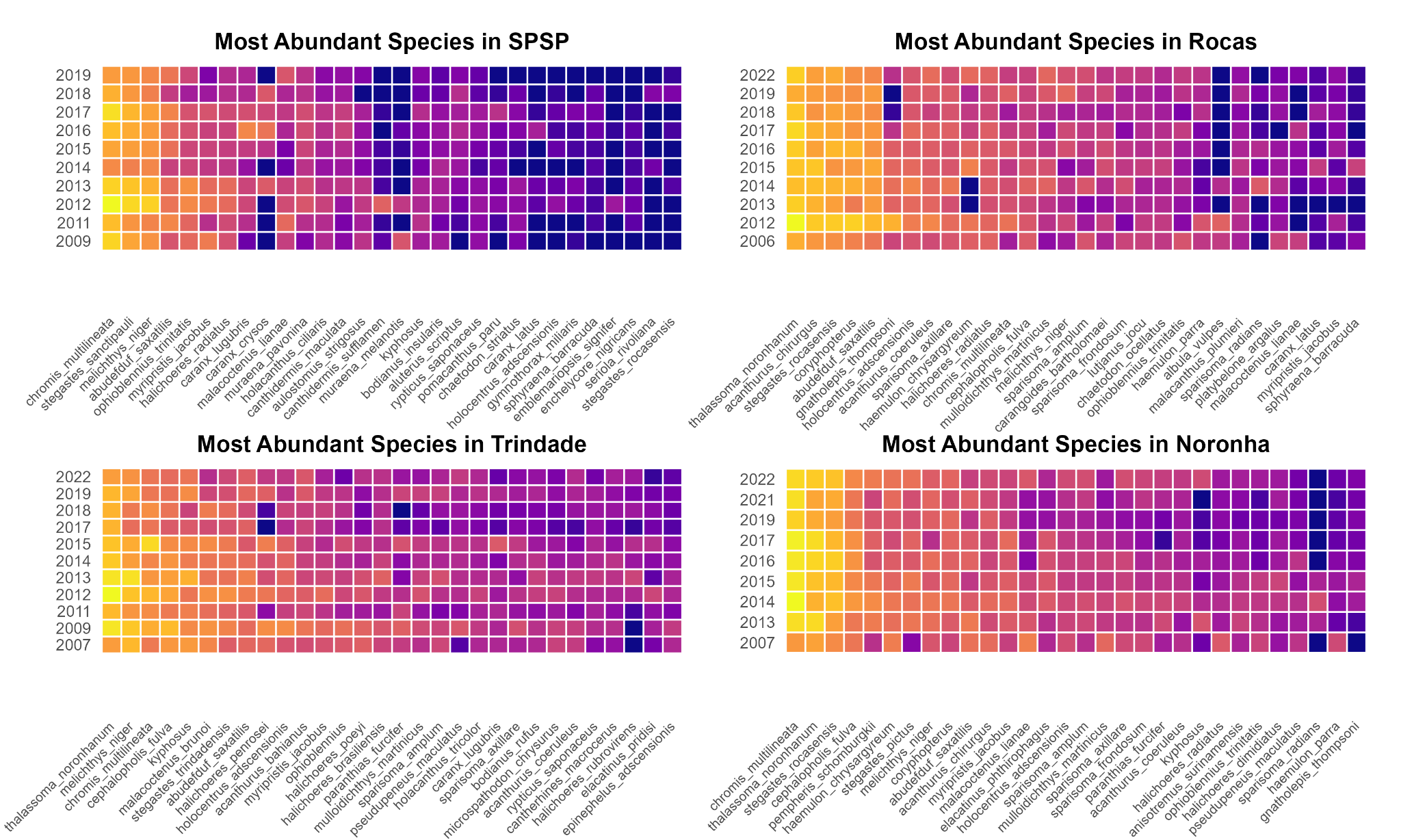


Figure SM 2- Heatmaps of the Abundance of Species (30 most abundant) in all islands over the years.


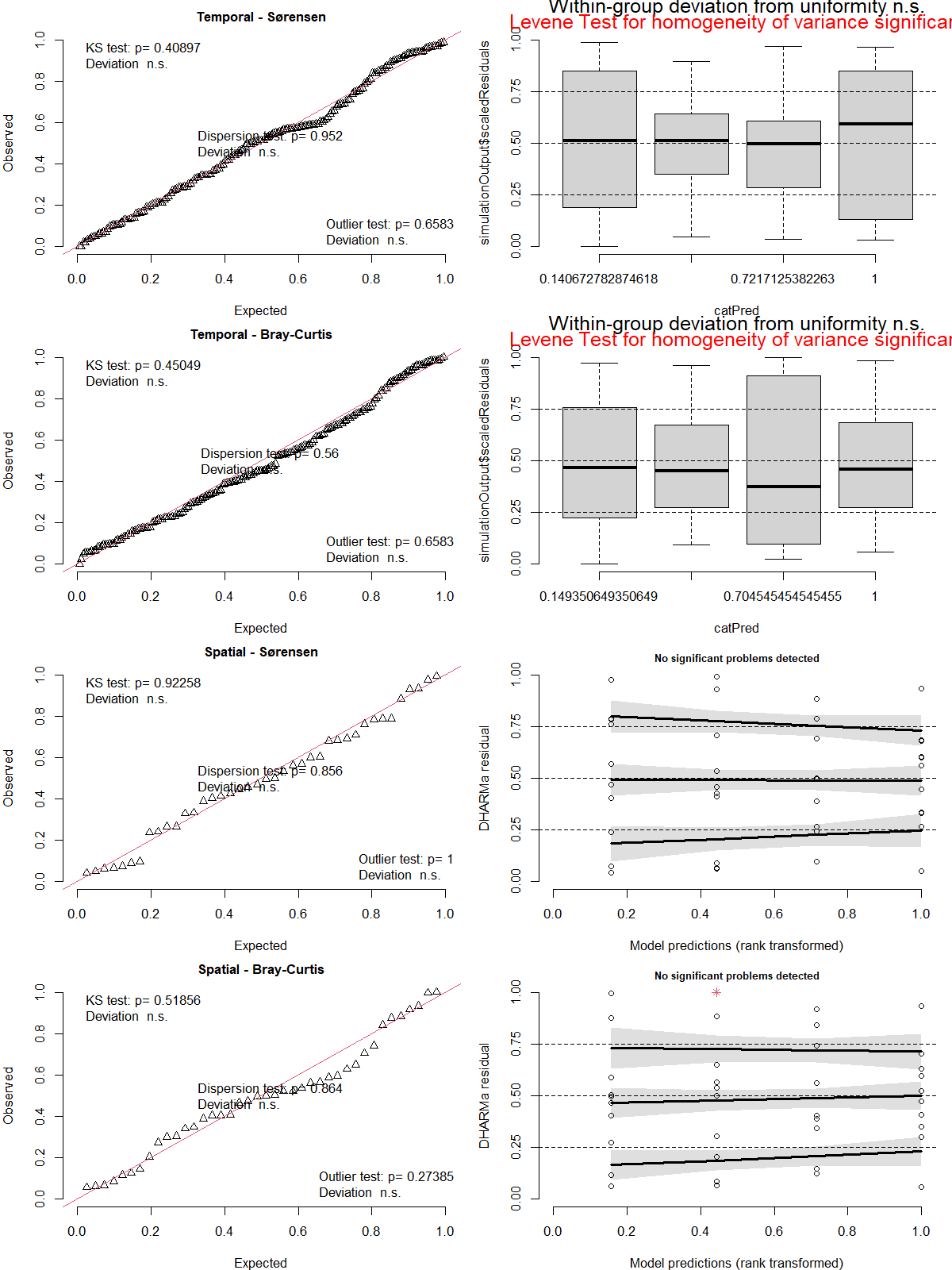


Figure SM 3- DHARMa residuals for the GLMMs (Temporal and Spatial, Sørensen and Bray-Curtis)

Table SM 3 Temporal and Spatial Sørensen and Bray-Curtis GLMMs (Significance codes: p < 0 ‘***’, p<0.001 ‘**’, p<0.01 ‘**’, p<0.05 ‘.’)

| Component | Term | Estimate | Std.Error | Z-Value | p-value | AIC | BIC | logLik | Deviance |
| --- | --- | --- | --- | --- | --- | --- | --- | --- | --- |
| Temporal Sørensen | Intercept | -1.254904 | 0.014598 | -85.97 | 2e-16*** | -701.8 | -682.6 | 356.9 | -713.8 |
|  | Distance | 0.009744 | 0.014976 | 0.65 | 0.51528 |  |  |  |  |
|  | Isolation | -0.046125 | 0.017100 | -2.70 | 0.00699** |  |  |  |  |
|  | Area | 0.073297 | 0.016516 | 4.44 | 9.09e-06*** |  |  |  |  |
| Temporal Bray-Curtis | Intercept | -1.22294 | 0.02094 | -58.39 | 2e-16*** | -567.9 | -548.7 | 290 | -579.9 |
|  | Distance | 0.09331 | 0.02128 | 4.39 | 1.16e-05*** |  |  |  |  |
|  | Isolation | -0.10563 | 0.02446 | -4.32 | 1.57e-05*** |  |  |  |  |
|  | Area | 0.08752 | 0.02394 | 3.66 | 0.000256*** |  |  |  |  |
| Spatial Sørensen | Intercept | -0.23376 | 0.03994 | -5.852 | 4.85e-09*** | -98.0 | -87.9 | 55.0 | -110.0 |
|  | Distance | 0.07516 | 0.04406 | 1.706 | 0.0880 |  |  |  |  |
|  | Isolation | -0.22178 | 0.05001 | -4.435 | 9.2e-06*** |  |  |  |  |
|  | Area | 0.09432 | 0.05186 | 1.819 | 0.0689* |  |  |  |  |
| Spatial Bray-Curtis | Intercept | -0.17296 | 0.04361 | -3.966 | 7.32e-05*** | -90.4 | -80.3 | 51.2 | -102.40 |
|  | Distance | 0.04047 | 0.04812 | 0.841 | 0.841 |  |  |  |  |
|  | Isolation | -0.25452 | 0.05457 | -4.664 | 3.11e-06*** |  |  |  |  |
|  | Area | 0.03063 | 0.05665 | 0.541 | 0.589 |  |  |  |  |

Table SM 4 Permutational Multivariate Analysis of Variance and Multivariate homogeneity of groups dispersions (betadisper) for reef fish assemblages among years within each island. (Significance codes: p < 0 ‘***’, p<0.001 ‘**’, p<0.01 ‘**’, p<0.05 ‘.’).

| Island | Bray-Curtis PERMANOVA | Jaccard PERMANOVA | Bray-Curtis Dispersion | Jaccard Dispersion |
| --- | --- | --- | --- | --- |
| Rocas | R²= 0.11*** | R²= 0.10*** | F= 4.50 | F= 3.56 |
| Noronha | R²= 0.16*** | R²= 0.79*** | F= 4.08 | F= 2.08 |
| SPSP | R²= 0.20*** | R²= 0.14*** | F= 8.43 | F= 6.71 |
| Trindade | R²= 0.13*** | R²= 0.09*** | F= 3.30 | F= 3.03 |

Table SM 5 Mean values of Beta diversity and its components per island and year.

| Island | Year | Sørensen | Simpson | Nestedness | Bray-Curtis | Balanced | Gradient |
| --- | --- | --- | --- | --- | --- | --- | --- |
| Noronha | 2007 | 0,48458498 | 0,384466403 | 0,100197628 | 0,572371542 | 0,465177866 | 0,106916996 |
| Noronha | 2013 | 0,494426877 | 0,401185771 | 0,092332016 | 0,482055336 | 0,368972332 | 0,113596838 |
| Noronha | 2014 | 0,533833992 | 0,463833992 | 0,069881423 | 0,522806324 | 0,443478261 | 0,079367589 |
| Noronha | 2015 | 0,468774704 | 0,421343874 | 0,04687747 | 0,453913043 | 0,388893281 | 0,064822134 |
| Noronha | 2016 | 0,523675889 | 0,458735178 | 0,065375494 | 0,463280632 | 0,385296443 | 0,077391304 |
| Noronha | 2017 | 0,448023715 | 0,372134387 | 0,075494071 | 0,41055336 | 0,337114625 | 0,073557312 |
| Noronha | 2019 | 0,406837945 | 0,333083004 | 0,073992095 | 0,406837945 | 0,333083004 | 0,073992095 |
| Noronha | 2021 | 0,447233202 | 0,359683794 | 0,088023715 | 0,499565217 | 0,415098814 | 0,084268775 |
| Noronha | 2022 | 0,564940711 | 0,471778656 | 0,093913043 | 0,562490119 | 0,477865613 | 0,084268775 |
| Rocas | 2006 | 0,435454545 | 0,340474308 | 0,094624506 | 0,498972332 | 0,372055336 | 0,127470356 |
| Rocas | 2012 | 0,415652174 | 0,285652174 | 0,130474308 | 0,46229249 | 0,345770751 | 0,115849802 |
| Rocas | 2013 | 0,343201581 | 0,235573123 | 0,107628458 | 0,36916996 | 0,255573123 | 0,113557312 |
| Rocas | 2014 | 0,353438735 | 0,26972332 | 0,082687747 | 0,424545455 | 0,340158103 | 0,084466403 |
| Rocas | 2015 | 0,611936759 | 0,20513834 | 0,406719368 | 0,647193676 | 0,249525692 | 0,39743083 |
| Rocas | 2016 | 0,467588933 | 0,363083004 | 0,104901186 | 0,476561265 | 0,414782609 | 0,061778656 |
| Rocas | 2017 | 0,405573123 | 0,339802372 | 0,065731225 | 0,41027668 | 0,336403162 | 0,073399209 |
| Rocas | 2018 | 0,352806324 | 0,244466403 | 0,108379447 | 0,362411067 | 0,275652174 | 0,086996047 |
| Rocas | 2019 | 0,424031621 | 0,359802372 | 0,063754941 | 0,481857708 | 0,412332016 | 0,069683794 |
| Rocas | 2022 | 0,515177866 | 0,286561265 | 0,22798419 | 0,544545455 | 0,297944664 | 0,246916996 |
| SpSp | 2009 | 0,466996047 | 0,315889328 | 0,152055336 | 0,539525692 | 0,332055336 | 0,208221344 |
| SpSp | 2011 | 0,243913043 | 0,146798419 | 0,09687747 | 0,272173913 | 0,171225296 | 0,101581028 |
| SpSp | 2012 | 0,34229249 | 0,267272727 | 0,075533597 | 0,351818182 | 0,279881423 | 0,072173913 |
| SpSp | 2013 | 0,319802372 | 0,22972332 | 0,090158103 | 0,331936759 | 0,250039526 | 0,081778656 |
| SpSp | 2014 | 0,30055336 | 0,220395257 | 0,080316206 | 0,303003953 | 0,227193676 | 0,075731225 |
| SpSp | 2015 | 0,333636364 | 0,248577075 | 0,085770751 | 0,350632411 | 0,227826087 | 0,122766798 |
| SpSp | 2016 | 0,384980237 | 0,273675889 | 0,112252964 | 0,426916996 | 0,300671937 | 0,126442688 |
| SpSp | 2017 | 0,394426877 | 0,297075099 | 0,097905138 | 0,36256917 | 0,289960474 | 0,072055336 |
| SpSp | 2018 | 0,387272727 | 0,300869565 | 0,087826087 | 0,346324111 | 0,278537549 | 0,067747036 |
| SpSp | 2019 | 0,256245059 | 0,203636364 | 0,05256917 | 0,252371542 | 0,205375494 | 0,046600791 |
| Trindade | 2007 | 0,474703557 | 0,391699605 | 0,082490119 | 0,483043478 | 0,405928854 | 0,077114625 |
| Trindade | 2009 | 0,521027668 | 0,420395257 | 0,099802372 | 0,538695652 | 0,424071146 | 0,114545455 |
| Trindade | 2011 | 0,510158103 | 0,407905138 | 0,103083004 | 0,531185771 | 0,436640316 | 0,094031621 |
| Trindade | 2012 | 0,405810277 | 0,328735178 | 0,076007905 | 0,408537549 | 0,321462451 | 0,086916996 |
| Trindade | 2013 | 0,523794466 | 0,388577075 | 0,1356917 | 0,508458498 | 0,380671937 | 0,127509881 |
| Trindade | 2014 | 0,536640316 | 0,44972332 | 0,08715415 | 0,551264822 | 0,454189723 | 0,096837945 |
| Trindade | 2015 | 0,537509881 | 0,390158103 | 0,147470356 | 0,530118577 | 0,368458498 | 0,161699605 |
| Trindade | 2017 | 0,470711462 | 0,36743083 | 0,103952569 | 0,498379447 | 0,387549407 | 0,110909091 |
| Trindade | 2018 | 0,48284585 | 0,3843083 | 0,098932806 | 0,518339921 | 0,409367589 | 0,108853755 |
| Trindade | 2019 | 0,614743083 | 0,556719368 | 0,058379447 | 0,635256917 | 0,562687747 | 0,072332016 |
| Trindade | 2022 | 0,520909091 | 0,433794466 | 0,087391304 | 0,524150198 | 0,415731225 | 0,108379447 |

Table SM 6 Variance-inflation factors (VIF) values.

| Variables | Spatial VIF values | Temporal VIF values |
| --- | --- | --- |
| Isolation | 1.503 | 1.304 |
| Distance | 1.210 | 1.068 |
| Area | 1.681 | 1.323 |

Table SM 7 Temporal and Spatial Sørensen and Bray-Curtis GLMMs using Logit transform with Gaussian (Significance codes: p < 0 ‘***’, p<0.001 ‘**’, p<0.01 ‘**’, p<0.05 ‘.’)

| Component | Term | Estimate | Std.Error | T-Value | p-value |
| --- | --- | --- | --- | --- | --- |
| Temporal Sørensen | Intercept | -1.263e+00 | 2.686e-02 | -47.01 | 2.2e-16 *** |
|  | Isolation | -1.903e-04 | 8.082e-05 | -2.35 | 0.019 * |
|  | Area | 1.053e-03 | 2.352e-04 | 4.47 | 1.34e-05 |
|  | Distance | 2.436e-05 | 3.099e-05 | 0.78 | 0.432 |
| Temporal Bray-Curtis | Intercept | -1.351e+00 | 4.712e-02 | -28.68 | 2.2e-16 *** |
|  | Isolation | -4.453e-04 | 1.038e-04 | -4.28 | 2.97e-05 *** |
|  | Area | 1.277e-03 | 4.060e-04 | 3.14 | 0.001 ** |
|  | Distance | 2.341e-04 | 4.767e-05 | 4.91 | 2.04e-06 *** |
| Spatial Sørensen | Intercept | -0.2391 | 0.1908 | -1.25 | 0.2176 |
|  | Isolation | -0.0009 | 0.0002 | -3.94 | 0.0003 *** |
|  | Area | 0.0075 | 0.0046 | 1.61 | 0.1146 |
|  | Distance | 0.0001 | 0.0001 | 1.44 | 0.1564 |
| Spatial Bray-Curtis | Intercept | -00157 | 0.1970 | -0.08 | 0.9367 |
|  | Isolation | -0.0010 | 0.0002 | -4.09 | 0.0002 *** |
|  | Area | 0.0024 | 0.0050 | 0.48 | 0.6300 |
|  | Distance | 0.0001 | 0.0001 | 0.75 | 0.4545 |

Table SM8- Kruskal-Wallis test Sørensen and Bray-Curtis and its components (Temporal and Spatial), as well for Species pool and Transect Richness, using Hellinger transformed data.

| Temporal Dissimilarity | | | | | | | | | |
| --- | --- | --- | --- | --- | --- | --- | --- | --- | --- |
| Metric | group1 | group2 | n1 | n2 | statistic | p | p.adj | p.adj.signif | p.adj.FDR.global |
| Bray-Curtis | Noronha | Rocas | 36 | 45 | -0,95740372 | 0,33836352 | 0,33836352 | ns | 0,380658959 |
| Bray-Curtis | Noronha | SpSp | 36 | 45 | -2,4274144 | 0,01520688 | 0,01520688 | * | 0,027361652 |
| Bray-Curtis | Noronha | Trindade | 36 | 55 | 3,43384046 | 0,00059509 | 0,00059509 | *** | 0,001785283 |
| Bray-Curtis | Rocas | SpSp | 45 | 45 | -1,55918177 | 0,11895336 | 0,11895336 | ns | 0,15294004 |
| Bray-Curtis | Rocas | Trindade | 45 | 55 | 4,72736042 | 2,2746E-06 | 2,2746E-06 | **** | 1,22396E-05 |
| Bray-Curtis | SpSp | Trindade | 45 | 55 | 6,36264406 | 1,9831E-10 | 1,9831E-10 | **** | 3,56958E-09 |
| Balanced | Noronha | Rocas | 36 | 45 | -2,61661767 | 0,00888057 | 0,00888057 | ** | 0,01776115 |
| Balanced | Noronha | SpSp | 36 | 45 | -3,24919614 | 0,00115732 | 0,00115732 | ** | 0,002975957 |
| Balanced | Noronha | Trindade | 36 | 55 | 1,66905542 | 0,0951064 | 0,0951064 | ns | 0,131685781 |
| Balanced | Rocas | SpSp | 45 | 45 | -0,67095078 | 0,50225188 | 0,50225188 | ns | 0,531796111 |
| Balanced | Rocas | Trindade | 45 | 55 | 4,69091184 | 2,7199E-06 | 2,7199E-06 | **** | 1,22396E-05 |
| Balanced | SpSp | Trindade | 45 | 55 | 5,39461096 | 6,8672E-08 | 6,8672E-08 | **** | 6,1805E-07 |
| Gradient | Noronha | Rocas | 36 | 45 | 2,76835783 | 0,00563396 | 0,00563396 | ** | 0,012676401 |
| Gradient | Noronha | SpSp | 36 | 45 | 1,16747122 | 0,24302012 | 0,24302012 | ns | 0,29162415 |
| Gradient | Noronha | Trindade | 36 | 55 | 3,46121816 | 0,00053774 | 0,00053774 | *** | 0,001785283 |
| Gradient | Rocas | SpSp | 45 | 45 | -1,69799667 | 0,08950839 | 0,08950839 | ns | 0,131685781 |
| Gradient | Rocas | Trindade | 45 | 55 | 0,61191166 | 0,54059621 | 0,54059621 | ns | 0,540596208 |
| Gradient | SpSp | Trindade | 45 | 55 | 2,39278559 | 0,01672101 | 0,01672101 | * | 0,027361652 |
| Spatial Dissimilarity | | | | | | | | | |
| Metric | Noronha | Rocas | 2277 | 2530 | -13,7983124 | 2,6089E-43 | 2,6089E-43 | **** | 5,2178E-43 |
| Bray-Curtis | Noronha | SpSp | 2277 | 2530 | -35,3842247 | 2,986E-274 | 2,986E-274 | **** | 5,374E-273 |
| Bray-Curtis | Noronha | Trindade | 2277 | 2783 | -20,5296328 | 1,1706E-93 | 1,1706E-93 | **** | 5,2675E-93 |
| Bray-Curtis | Rocas | SpSp | 2530 | 2530 | -22,1774167 | 5,674E-109 | 5,674E-109 | **** | 3,405E-108 |
| Bray-Curtis | Rocas | Trindade | 2530 | 2783 | -6,60858066 | 3,8802E-11 | 3,8802E-11 | **** | 6,3495E-11 |
| Bray-Curtis | SpSp | Trindade | 2530 | 2783 | 16,0907291 | 2,9633E-58 | 2,9633E-58 | **** | 7,6198E-58 |
| Bray-Curtis | Noronha | Rocas | 2277 | 2530 | -13,4559596 | 2,8403E-41 | 2,8403E-41 | **** | 5,1125E-41 |
| Balanced | Noronha | SpSp | 2277 | 2530 | -33,3394538 | 1,036E-243 | 1,036E-243 | **** | 9,322E-243 |
| Balanced | Noronha | Trindade | 2277 | 2783 | -18,551336 | 7,955E-77 | 7,955E-77 | **** | 2,3865E-76 |
| Balanced | Rocas | SpSp | 2530 | 2530 | -20,4283484 | 9,3615E-93 | 9,3615E-93 | **** | 3,3701E-92 |
| Balanced | Rocas | Trindade | 2530 | 2783 | -4,93353979 | 8,0753E-07 | 8,0753E-07 | **** | 1,1181E-06 |
| Balanced | SpSp | Trindade | 2530 | 2783 | 15,9755414 | 1,892E-57 | 1,892E-57 | **** | 4,2571E-57 |
| Balanced | Noronha | Rocas | 2277 | 2530 | 1,72264108 | 0,08495345 | 0,08495345 | ns | 0,08995071 |
| Gradient | Noronha | SpSp | 2277 | 2530 | -3,72348873 | 0,00019649 | 0,00019649 | *** | 0,00025263 |
| Gradient | Noronha | Trindade | 2277 | 2783 | -1,00670359 | 0,31407724 | 0,31407724 | ns | 0,31407724 |
| Gradient | Rocas | SpSp | 2530 | 2530 | -5,59536648 | 2,2016E-08 | 2,2016E-08 | **** | 3,3023E-08 |
| Gradient | Rocas | Trindade | 2530 | 2783 | -2,8470795 | 0,00441223 | 0,00441223 | ** | 0,00496376 |
| Gradient | SpSp | Trindade | 2530 | 2783 | 2,87996068 | 0,00397725 | 0,00397725 | ** | 0,0047727 |
| Gradient | Noronha | Rocas | 2277 | 2530 | -13,7983124 | 2,6089E-43 | 2,6089E-43 | **** | 5,2178E-43 |
